# Community Knowledge, Risk Perception and Health-Seeking Behaviour Toward Rabies in Ghana: One Health Implications

**DOI:** 10.3390/tropicalmed11030063

**Published:** 2026-02-26

**Authors:** Prince Kyere Dwaah, Nana Yaa Awua-Boateng, Sylvia Afriyie Squire, Ernest Osei, David Kando, Rogermilla Enam Dunu, Daniel Nartey, Helen Djang-Fordjour, Patience Edze

**Affiliations:** 1 Disease Investigation Farm/Regional Veterinary Laboratory, Veterinary Service Directorate, Techiman P.O. Box 122, Ghana; 2 Public Health Education Department, Faculty of Environment and Public Health Education, Akenten Appiah-Menka University of Skill Training and Entrepreneurial Development, Kumasi P.O. Box 1277, Ghana; nyawua-boateng@aamusted.edu.gh (N.Y.A.-B.); eosei@aamusted.edu.gh (E.O.); krogermilla99@gmail.com (R.E.D.); patienceedze9@gmail.com (P.E.); 3 Council for Scientific and Industrial Research (CSIR)—Animal Research Institute, CSIR College of Science and Technology, Accra P.O. Box AH 20, Ghana; ssquire@csir.org.gh; 4 Ghana Health Service, Municipal Health Directorate, Techiman 035 25, Ghana; kandodavid@yahoo.com; 5 Veterinary Service Directorate, Tema Metropolis Veterinary Service, Tema P.O. Box M161, Ghana; danielnartey2110@gmail.com; 6 Department of Animal Science and Fisheries, School of Agriculture and Environmental Science, Evangelical Presbyterian University College, Ho P.O. Box HP 678, Ghana; 7 Department of Agriculture, Faculty of Applied Science and Technology, Sunyani Technical University, Sunyani P.O. Box 206, Ghana; 8 Krowor Municipal Assembly, Environmental Health Officer, Accra 0524, Ghana

**Keywords:** rabies, Ghana, community knowledge, risk perception, health-seeking behaviour, dog vaccination, One Health

## Abstract

Rabies remains a zoonotic public health problem in Ghana despite the availability of effective preventive measures, including mass dog vaccination and timely post-exposure prophylaxis (PEP). We conducted a community-based cross-sectional mixed-methods study between June and December 2025 to assess rabies-related knowledge, risk perception, health-seeking behaviour following dog bites, and dog vaccination practices within a One Health framework. Structured face-to-face interviews were conducted with 450 adults from selected urban and rural communities in the Greater Accra, Ashanti, and Bono East regions, supplemented by focus group discussions. Quantitative data were analysed using descriptive statistics, chi-square tests, and multivariable logistic regression. Overall, 68% of respondents had heard of rabies; however, detailed knowledge of transmission and prevention was limited, with 189 (42.0%) correctly identifying dogs as the main source of transmission. Following suspected exposure, 162 (36.0%) reported using home remedies or traditional treatments. Dog vaccination coverage was 31.1%, below the level required to interrupt transmission. Educational level, place of residence, and prior dog-bite exposure were significantly associated with rabies knowledge, health-seeking behaviour, and vaccination practices (*p* < 0.05). This study provides updated evidence on community rabies knowledge, risk perception, and preventive practices, highlighting behavioural and structural gaps that may hinder effective control in Ghana.

## 1. Introduction

Rabies is an acute viral zoonosis that is almost invariably fatal once clinical symptoms appear, but is entirely preventable through timely post-exposure prophylaxis (PEP) and sustained mass dog vaccination [1,2]. Globally, dog-mediated rabies causes an estimated 59,000 human deaths annually, with the greatest burden in low- and middle-income countries in Africa and Asia [2,3,4]. Domestic dogs account for the majority of human exposures in these regions.

Knowledge, attitude, and practice (KAP) surveys are widely used to assess community understanding, perceptions, and behaviours related to health and disease. First developed in the 1950s for family planning and population studies, KAP surveys have since been applied broadly in public health to inform programme design and evaluation [5,6]. KAP surveys generate population-representative data describing what people know about a disease, how they perceive associated risks, and how they respond in practice. In the context of rabies, KAP surveys identify behavioural and social factors that influence exposure risk, health-seeking behaviour, and participation in dog vaccination programmes [7,8].

In Ghana, rabies remains endemic despite available preventive tools. Retrospective analyses indicate that suspected and confirmed human rabies cases are reported annually; however, underreporting is likely, particularly in rural areas with limited diagnostic capacity and health access [9,10]. Domestic dogs constitute the principal reservoir, and free-roaming populations are common in both urban and rural communities [9,10,11].

Rabies prevention and control in Ghana involve both the veterinary and public health sectors. The Veterinary Services Directorate conducts dog vaccination campaigns and passive surveillance, while the Ghana Health Service manages dog-bite cases and provides PEP at selected facilities. Access to PEP is variable, and delays in seeking care following dog bites have been documented [10,11]. Dog vaccination coverage remains inconsistent and below the ≥70% threshold required to interrupt transmission [4].

Evidence from Ghana and other endemic settings suggests that community knowledge, risk perception, and health-seeking behaviour strongly influence rabies outcomes [6,9,10,11]. Misconceptions about transmission, reliance on traditional remedies, and underestimation of bite-related risk are commonly reported, particularly in rural and peri-urban areas [10,11,12]. These behaviours often interact with structural constraints, such as access to veterinary and health services.

Mass dog vaccination is the cornerstone of rabies elimination and the most effective strategy to prevent human rabies deaths [4,13]. However, coverage in Ghana remains low [9,10,11]. Understanding community-level determinants of dog vaccination and responses to potential exposure is therefore essential. This study assessed rabies knowledge, risk perception, health-seeking behaviour, and dog vaccination practices in selected urban and rural communities in Ghana, using a One Health framework to inform integrated control strategies.

## 2. Materials and Methods

### 2.1. Study Design and Study Area

A community-based cross-sectional mixed-methods study was conducted between June and December 2025 in selected districts of Greater Accra, Ashanti, and Bono East regions. These regions were purposively selected to capture variation in urbanization, dog ownership patterns, access to veterinary and health services, and reported dog-bite incidence [9,10]. Greater Accra is predominantly urban, with better access to services, while parts of Ashanti and Bono East include rural communities with more free-roaming dogs and limited health and veterinary access.

### 2.2. Study Population and Eligibility Criteria

Adults aged ≥18 years who had lived in the community for ≥6 months were eligible. The quantitative survey primarily focused on households caring for at least one dog. Individuals unable or unwilling to provide consent were excluded.

### 2.3. Sample Size Determination and Sampling Strategy

Sample size was calculated using the single population proportion formula [7,8,10]: $n=\frac{Z^{2}\;p(1-p)}{d^{2}},\;$where n = sample size, Z = 1.96 for 95% confidence, p = 0.5 (assumed prevalence), and d = 0.05 (margin of error). To account for non-response, $n_{final}=\frac{n}{1-r}$, where r is the anticipated non-response rate. The final target was 450 respondents.

### 2.4. Data Collection Instruments and Procedures

Quantitative data were collected using a structured questionnaire adapted from validated rabies KAP tools [7,8,10] and pretested in a non-study community (File S1 2.0). The data captured socio-demographics, rabies knowledge, risk perception, dog-bite management, health-seeking behaviour, and dog vaccination history.

Focus group discussions (6–8 participants each) explored community beliefs about rabies, treatment pathways, and barriers to vaccination and PEP.

### 2.5. Data Management and Analysis

Quantitative data were analysed using SPSS v26. Descriptive statistics summarized respondent characteristics, knowledge, risk perception, and practices. Knowledge scores were categorized as adequate or inadequate [7,8]. Chi-square tests and multivariable logistic regression identified predictors of knowledge, health-seeking behaviour, and dog vaccination. Significance was set at *p* < 0.05.

Qualitative data were transcribed and analysed thematically to contextualize quantitative findings.

Multivariable logistic regression models were fitted to identify factors independently associated with rabies knowledge, health-seeking behaviour following dog bites, and dog vaccination status. Variables with *p* < 0.20 in bivariate analysis and those considered biologically and contextually relevant based on previous literature were included in the models.

Potential confounding variables considered included educational level, place of residence (urban/rural), dog confinement status, and prior dog-bite exposure. Model results are presented as adjusted odds ratios (aORs) with corresponding 95% confidence intervals (CIs). Statistical significance was set at *p* < 0.05.

## 3. Results

### 3.1. Socio-Demographic Characteristics

Of 450 respondents, 46.7% were urban and 53.3% rural. Education ranged from no formal schooling to tertiary level; lower education and free-roaming dog management were more common in rural communities (Table 1).

### 3.2. Community Knowledge of Rabies

Overall awareness was higher in urban areas, with 42.0% identifying dogs as the main transmission source. Knowledge gaps were greatest among rural residents with no formal education (Table 2).

### 3.3. Risk Perception, Health-Seeking Behaviour and PEP Utilization

**Figure 1:**
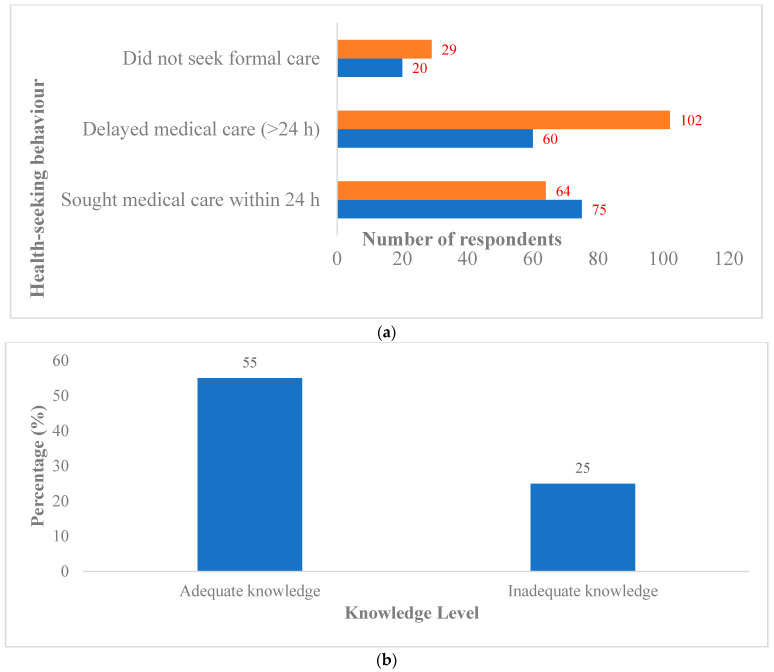
(**a**) Health-seeking behaviour following dog-bite exposure by residence. Note: Orange → Rural and Blue → Urban. (**b**) Seeking medical care after dog bite. Note: Differences in delayed care between urban and rural respondents were statistically significant (χ^2^ test, *p* < 0.05).

In total, 58% perceived dog bites as potentially dangerous. Rural respondents were more likely to consider minor bites low risk and delay formal care; 36.0% used home remedies or traditional treatments; only 31.0% sought care within 24 h (Table 3, Figure 1).

### 3.4. Qualitative Findings from Focus Group Discussions

Focus group discussions revealed important contextual insights into community perceptions and practices related to rabies that complemented the quantitative findings. Participants generally demonstrated awareness that rabies is associated with dog bites; however, their detailed understanding of disease severity and fatal outcomes was limited. Minor bites and scratches, particularly from familiar or owned dogs, were frequently perceived as low risk and not requiring urgent medical attention.

Many participants reported relying on home remedies and traditional treatments, including herbal preparations and local wound dressings, as first-line responses to dog bites. This practice was particularly common in rural communities and was often attributed to long-standing cultural beliefs, perceived effectiveness of traditional medicine, and limited access to formal health facilities.

Barriers to timely post-exposure prophylaxis (PEP) identified during the discussions included perceived high cost of treatment, distance to health facilities that provide PEP, lack of information on where PEP services are available, and underestimation of the urgency of seeking care.

Regarding dog vaccination, participants cited irregular vaccination campaigns, cost of vaccination, poor access to veterinary services, and the challenge of managing free-roaming dogs as major constraints. Some dog owners expressed uncertainty about the benefits of vaccination or reported that dogs were difficult to restrain for vaccination. These findings provide a qualitative context for the observed low vaccination coverage and delayed health-seeking behaviour reported in the quantitative survey.

### 3.5. Dog Vaccination Coverage and Geographic Differences

**Figure 2:**
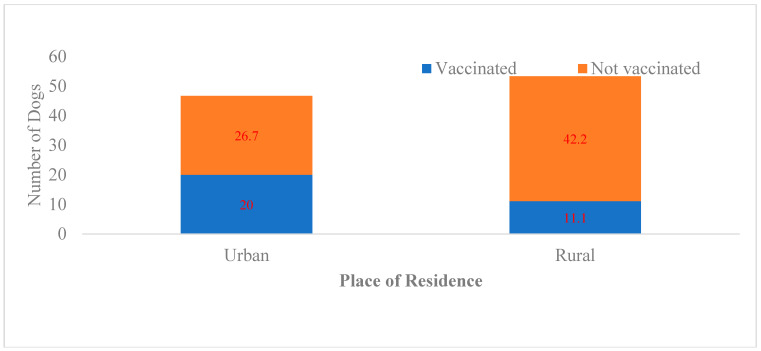
Dog vaccination coverage by place of residence. Note: Orange → Not vaccinated and Blue → Vaccinated. Vaccination coverage differed significantly by residence.

Overall coverage was 31.1%, higher in urban households (42.9%) than in rural households (20.8%) (Figure 2). Free-roaming dogs were less likely to be vaccinated. Dog confinement, urban residence, and higher owner education were positively associated with vaccination (Table 4).

## 4. Discussion

This study provides updated evidence on community-level knowledge, risk perception, health-seeking behaviour, and dog vaccination practices in selected urban and rural settings in Ghana. The findings indicate that rabies control in these communities is constrained primarily by behavioural and structural factors rather than by the absence of effective preventive tools. Although general awareness of rabies was moderate, detailed understanding of transmission pathways, disease severity, and prevention remained limited, particularly among respondents with lower educational attainment and those residing in rural areas [5,7,10,11].

Only 42.0% of respondents correctly identified dogs as the main source of rabies transmission, and fewer than half recognized bite wounds as high-risk exposures. Similar discrepancies between general awareness and accurate knowledge have been reported in previous studies conducted in Ghana and other rabies-endemic settings, including Tanzania, Ethiopia, and Nigeria [7,8,9,11]. These findings suggest that awareness alone may be insufficient to promote appropriate preventive behaviour, especially in contexts where dog bites are frequent and socially normalized.

The qualitative findings from the focus group discussions provide important explanatory insight into these quantitative patterns. Participants commonly perceived minor bites or scratches, particularly those inflicted by familiar or owned dogs, as low risk, thereby reducing the perceived urgency of seeking formal medical care. This normalization of exposure risk helps explain the observed delays in health-seeking behaviour and the low uptake of timely PEP. Comparable perceptions have been documented in other rabies-endemic regions of sub-Saharan Africa and Asia, where routine interaction with dogs often leads to underestimation of bite-related risk [8,11,12].

Reliance on home remedies and traditional treatments following dog bites was frequently reported, especially in rural communities. While these practices were rooted in cultural beliefs, they were also shaped by structural constraints, including distance to health facilities, uncertainty regarding the availability of PEP services, and perceived treatment costs. Similar interactions between belief systems and access barriers have been reported in Ghana, Uganda, and Ethiopia, where both factors contribute to delayed PEP initiation [2,9,10,14]. These findings indicate that interventions aimed at improving health-seeking behaviour must address both sociocultural norms and health system accessibility.

The low uptake of PEP observed in this study (26.7%) further reflects these combined behavioural and structural challenges. Rural residence was significantly associated with delayed PEP initiation, consistent with previous studies showing that limited geographic access, irregular vaccine availability, and health system constraints disproportionately affect rural populations [2,9,10,14]. Despite the inclusion of rabies PEP within national public health systems, these barriers continue to place rural communities at heightened risk.

Dog vaccination coverage in the study population was low (31.1%) and substantially below the ≥70% threshold required to interrupt rabies transmission in dog populations [4,13]. Free-roaming dogs accounted for the majority of unvaccinated animals, and dog confinement was strongly associated with vaccination status. Insights from the focus group discussions further suggest that low vaccination coverage reflects systemic challenges rather than unwillingness among dog owners. Participants highlighted irregular vaccination campaigns, limited access to veterinary services, cost considerations, and difficulties restraining free-roaming dogs as key barriers. Similar associations between dog management practices, owner education, and vaccination uptake have been reported in studies from Tanzania, Kenya, India, and Ghana [6,7,10,13].

The observed urban–rural disparities in vaccination coverage are consistent with earlier findings from Ghana, where urban dog owners generally have better access to veterinary services and vaccination campaigns [6,7,9]. Persistent structural limitations, including shortages of veterinary personnel and inconsistent outreach activities, continue to undermine sustained vaccination coverage, particularly in rural and peri-urban settings [9,11,13].

Overall, the integrated quantitative and qualitative findings highlight the interconnected behavioural and structural determinants sustaining rabies risk. Misconceptions about transmission, underestimation of bite severity, reliance on traditional remedies, and weak dog management practices interact with limited access to health and veterinary services to create conditions favourable for ongoing transmission [7,8,10,13,15]. Similar conclusions have been drawn in multi-country analyses, which emphasize that rabies elimination requires coordinated interventions targeting both human behaviour and dog populations [3,4,13].

This study reinforces the importance of integrated One Health approaches that combine community-centred education, improved access to timely PEP, strengthened and regularized dog vaccination campaigns, and enhanced coordination between veterinary and public health services [13,14]. Such approaches have demonstrated effectiveness in pilot rabies elimination programmes in Africa and Asia and are central to achieving the global goal of ending dog-mediated human rabies deaths by 2030 [3,4,13,14].

This study has several limitations. Restricting the quantitative survey primarily to dog-owning households may limit generalizability to the wider community. Data were self-reported and subject to recall and social desirability bias. The cross-sectional design precludes causal inference. Despite these limitations, the study provides important insights into behavioural and structural drivers of rabies risk in both urban and rural Ghanaian settings.

## 5. Conclusions

Rabies persists in Ghana due to social, behavioural, and structural factors. Limited knowledge, delayed health-seeking, reliance on traditional remedies, and low dog vaccination coverage contribute to sustained transmission, particularly in rural areas. Effective rabies control requires coordinated One Health interventions integrating community education, improved PEP access, and sustained mass dog vaccination.

## Figures and Tables

**Table 1:** Socio-demographic characteristics of survey respondents (n = 450).

Variable	Category	n (%)
Sex	Male	220 (48.9)
	Female	230 (51.1)
Residence	Urban	210 (46.7)
	Rural	240 (53.3)
Education level	No formal education	80 (17.8)
	Primary	120 (26.7)
	Secondary	150 (33.3)
	Tertiary	100 (22.2)
Dog ownership	Yes	270 (60.0)
	No	180 (40.0)

**Table 2:** Knowledge of rabies transmission and outcomes among respondents.

Knowledge Item	Correct Response n (%)
Identified dogs as the main transmission source	189 (42.0)
Recognized rabies as fatal after symptom onset	168 (37.3)
Identified bite wounds as high-risk exposure	202 (44.9)
Awareness of post-exposure prophylaxis	216 (48.0)

**Table 3:** Risk perception, dog-bite management, and health-seeking behaviour among respondents (n = 450).

Category	Subcategory/Practice	n (%)
Risk Perception	Considered dog bites potentially dangerous	261 (58.0)
	Perceived minor bites or scratches as low risk	189 (42.0)
Dog-Bite Management Practices	Washed wound with soap and water	180 (40.0)
	Used home remedies or traditional treatments	162 (36.0)
	No immediate wound care	108 (24.0)
Health-Seeking Behaviour	Sought formal medical care within 24 h	139 (31.0)
	Delayed medical care (>24 h)	162 (36.0)
	Did not seek formal care	49 (10.9)
Post-Exposure Prophylaxis (PEP) Utilization	Received PEP	120 (26.7)
	Did not receive PEP	330 (73.3)
Factors Associated with PEP Uptake	Higher knowledge score → timely PEP	*p* < 0.01
	Rural residence → delayed PEP	*p* < 0.05

**Table 4:** Dog vaccination coverage and associated factors.

Factor	Vaccinated % (n/N)	aOR (95% CI)	*p*-Value
Urban residence	42.9 (90/210)	2.85 (1.90–4.27)	<0.01
Rural residence	20.8(50/240)	Reference	
Confined dog	57.1 (80/140)	5.57 (3.40–9.12)	<0.01
Free-roaming dog	19.4 (60/310)	Reference	
Tertiary education	50.0 (50/100)	3.00 (1.65–5.45)	<0.05
No formal education	25.0 (20/80)	Reference	

## Data Availability

The datasets generated and/or analysed during the current study are available from the corresponding authors upon reasonable request.

## Supplementary Materials

The following supporting information can be downloaded at https://www.mdpi.com/article/10.3390/tropicalmed11030063/s1: File S1: Informed consent form for study participation and questionnaire. 1.0: Informed consent form for study participation. 2.0: Structured questionnaire used for the community survey on rabies knowledge, risk perception, health-seeking behaviour, and dog vaccination practices.

## Abbreviations

The following abbreviations are used in this manuscript:

CSIR	Council for Scientific and Industrial Research
FAO	Food and Agriculture Organization of the United Nations
FGD	Focus Group Discussion
FGDs	Focus Group Discussions
GHS	Ghana Health Service
KAP	Knowledge, Attitudes, and Practices
MMWR	Morbidity and Mortality Weekly Report
PEP	Post-Exposure Prophylaxis
SPSS	Statistical Package for the Social Sciences
UNICEF	United Nations Children’s Fund
WHO	World Health Organization
