# Supplementary material for: Community Knowledge, Risk Perception and Health-Seeking Behaviour Toward Rabies in Ghana: One Health Implications
Source: Trop Med Infect Dis. 2026 Feb 26;11(3):63. doi: 10.3390/tropicalmed11030063 (PMC13030535; doi:10.3390/tropicalmed11030063)
Supplement: Supplementary file 1 [file tropicalmed-11-00063-s001.zip › tropicalmed-4126273-supplementary.pdf]

SUPPLEMENTARY FILE

1.0 INFORMED CONSENT FORM FOR STUDY PARTICIPATION

Title of the Study:
Societal Knowledge, Risk Perception, and Health-Seeking Behaviour toward Rabies in Ghana: Implications for One Health Policy and Dog Vaccination Coverage

Principal Investigators:
[1 & 2] Prince Kyere Dwaah
[3] Helen Djang-Fordjour

Institutions:
[1] Veterinary Service Directorate, Disease Investigation Farm/Regional Veterinary Laboratory-Techiman, Ghana.

[2] University of Skill Training and Entrepreneurial Development, Asante Mampong Campus, Ghana.

[3] Sunyani Technical University, Sunyani, Ghana

Introduction
You are being invited to participate in a research study that aims to understand community knowledge, perceptions, and practices related to rabies, dog bites, and dog vaccination in Ghana. Before you decide whether to participate, you must understand why the research is being conducted and what your participation will involve. Please take time to listen carefully to the information provided and ask any questions you may have.

Purpose of the Study
The purpose of this study is to assess public knowledge of rabies, perceptions of risk associated with dog bites, health-seeking behaviour following exposure, and factors influencing dog vaccination practices. The findings will be used to inform public health and veterinary policies and improve rabies prevention and control strategies in Ghana.

Procedures
If you agree to participate, you will be asked to take part in an interview using a structured questionnaire. The questions will cover basic demographic information, knowledge of rabies, perceptions of dog-bite risk, actions taken after dog bites, and dog ownership and vaccination practices. The interview will take approximately 20-30 minutes. In some communities, participants may also be invited to join a group discussion to share general views and experiences related to rabies prevention.

Voluntary Participation
Your participation in this study is entirely voluntary. You are free to choose whether or not to take part. If you decide to participate, you may refuse to answer any question or withdraw from the study at any time without any penalty or loss of benefits to which you are otherwise entitled.

Risks and Discomforts
This study involves minimal risk. No physical procedures will be performed, and no biological samples will be collected. Some questions may involve recalling past experiences with dog bites or illness, which may cause mild discomfort. You are free to skip any question you do not wish to answer.

Benefits
There is no direct financial or material benefit to you for participating in this study. However, your participation will contribute to improving understanding of rabies prevention and may help inform future public health and veterinary interventions that benefit communities in Ghana.

Confidentiality and Anonymity
All information you provide will be kept strictly confidential. No names, addresses, or other personal identifiers will be recorded. Data will be anonymized and stored securely, and only the research team will have access to the information. Study results will be reported in aggregated form and will not identify any individual participant.

Consent for Publication
The study does not involve the collection of identifiable personal data, photographs, or audio-visual recordings for publication. The information collected will be used solely for research and academic publication purposes in anonymized form.

Compensation
You will not receive any payment or compensation for participating in this study.

Questions or Concerns
If you have any questions about the study or your rights as a participant, you may contact the principal investigator or the research team through the provided institutional channels.

Statement of Consent
I have been informed about the purpose, procedures, risks, and benefits of this study. I have had the opportunity to ask questions, and all my questions have been answered to my satisfaction. I understand that my participation is voluntary and that I may withdraw at any time without consequences. By agreeing verbally to participate, I confirm that I freely give my informed consent to take part in this study.

Participant's Consent: ☐ Yes ☐ No

Date: ……………………………………..

Name/Signature of Research Assistant: ……………………………………..

2.0 QUESTIONNAIRE

Title:

Societal Knowledge, Risk Perception, and Health-Seeking Behaviour toward Rabies in Ghana: Implications for One Health Policy and Dog Vaccination Coverage

Section A: Demographics

1. Respondent ID:
2. Age:
3. Sex: [ ] Male [ ] Female
4. Region: [ ] Bono East (Techiman) [ ] Northern (Savelugu) [ ] Greater Accra (Nungua)
5. Residence type: [ ] Urban [ ] Rural
6. Education level: [ ] No formal [ ] Primary [ ] Secondary [ ] Tertiary
7. Occupation:
8. Household size:

Section B: Dog Ownership and Management

9. Do you own a dog? [ ] Yes [ ] No
10. Number of dogs in the household:
11. Are your dogs confined or allowed to roam freely? [ ] Confined [ ] Free-roaming
12. Have your dogs been vaccinated against rabies? [ ] Yes [ ] No
13. If yes, year of last vaccination:
14. Are there stray dogs frequently present in your community? [ ] Yes [ ] No

Section C: Knowledge of Rabies

15. Have you heard of rabies? [ ] Yes [ ] No
16. What animals can transmit rabies?

[ ] Dogs [ ] Cats [ ] Bats [ ] Monkeys [ ] Others (specify)

17. How is rabies transmitted to humans?

[ ] Bite [ ] Scratch [ ] Saliva contact [ ] Others (specify)

18. Is rabies preventable? [ ] Yes [ ] No [ ] Not sure
19. Are you aware of Post-Exposure Prophylaxis (PEP) after a bite? [ ] Yes [ ] No
20. What are the symptoms of rabies in humans? (list)

Section D: Risk Perception

21. How serious do you perceive dog bites to be? [ ] Low [ ] Medium [ ] High

22. How likely do you think you or household members could get rabies if bitten by a dog? [ ] Unlikely [ ] Possible [ ] Likely

Section E: Health-Seeking Behaviour

23. If bitten by a dog, what would you do first?
[ ] Go to hospital/clinic [ ] Home treatment [ ] Traditional healer [ ] Others (specify)

24. Have you or any household member ever been bitten by a dog? [ ] Yes [ ] No

25. If yes, what actions were taken?
[ ] PEP at hospital [ ] Home treatment [ ] Traditional healer [ ] Nothing

26. Did the person bitten survive? [ ] Yes [ ] No [ ] N/A

Section F: Attitudes and Practices

27. Do you believe dog vaccination is necessary for public health? [ ] Yes [ ] No [ ] Not sure

28. Would you be willing to vaccinate your dog if free vaccination campaigns were available? [ ] Yes [ ] No [ ] Not sure

29. Do you kill or avoid stray dogs? [ ] Yes [ ] No

30. Do you inform authorities about suspected rabid dogs? [ ] Yes [ ] No

THANK YOU
